# Effects of venetoclax, a BCL2 inhibitor, in systemic chronic active Epstein-Barr virus disease

**DOI:** 10.1038/s41598-025-03719-9

**Published:** 2025-05-27

**Authors:** Ayaka Ohashi, Miwako Nishio, Mayumi Yoshimori, Kaoru Koike, Morito Kurata, Hayato Tamai, Ken-Ichi Imadome, Ayako Arai

**Affiliations:** 1 Department of Immunology and Parasitology, St. Marianna University School of Medicine, Kanagawa, Japan; 2 Department of Hematology and Biophysical Systems Analysis, Graduate School of Medical and Dental Sciences, Institute of Science Tokyo, Tokyo, Japan; 3 Center of Blood Transfusion and Cell Therapy, Institute of Science Tokyo Hospital, Tokyo, Japan; 4 Division of Integrated Facilities, Pathology, Institute of Science Tokyo, Tokyo, Japan; 5 Department of Hematology and Oncology, St. Marianna University School of Medicine, Kanagawa, Japan; 6 Department of Infectious diseases and Infection Control, Akiru Municipal Medical Center, Tokyo, Japan

**Keywords:** Venetoclax, BCL2, Systemic chronic active Epstein-Barr virus disease, sCAEBV, EBV, Cancer, Microbiology, Molecular medicine, Oncology

## Abstract

Systemic chronic active Epstein-Barr virus disease (sCAEBV) is a chemotherapy-resistant, EBV-positive T- or NK-cell lymphoproliferative disorder characterized by persistent systemic inflammation driven by the activation of EBV-infected cells. In this study, we explored BCL2, an anti-apoptotic factor implicated in various hematopoietic malignancies, as a potential therapeutic target for sCAEBV, focusing on the effects of its inhibitor, venetoclax. We confirmed BCL2 expression in EBV-positive T- and NK-cell lines and peripheral blood mononuclear cells (PBMCs) from sCAEBV patients using western blotting. Immunofluorescence staining further revealed BCL2 expression in EBV-infected cells within patient-derived PBMCs. Venetoclax treatment reduced the viability of EBV-positive cell lines and patient-derived PBMCs in a dose-dependent manner and induced apoptosis in these cells. Moreover, venetoclax suppressed the mRNA expression of the inflammatory cytokine IFN-γ in patient-derived PBMCs. To evaluate the in vivo effects of venetoclax, we utilized sCAEBV xenograft model generated by transplanting patient-derived PBMCs into NOD/Shi-scid/IL-2Rγnull mice. No engraftment of EBV-infected cells was observed in mice treated with venetoclax, whereas one out of three mice in the untreated group exhibited engraftment of EBV-positive cells and tumor formation. Venetoclax treatment showed an insignificant trend to reducing IFN-γ levels in peripheral blood in established xenograft models. To our knowledge, this is the first report to suggest that venetoclax exerts not only anti-tumor effects but also potential anti-inflammatory effects in sCAEBV. BCL2 represents a promising therapeutic target to address the two pathological characteristics of sCAEBV: malignancy and inflammation.

**Supplementary Information:**

The online version contains supplementary material available at 10.1038/s41598-025-03719-9.

## Introduction

Systemic chronic active Epstein-Barr virus disease (sCAEBV) is a lymphoproliferative disorder characterized by the activation and clonal proliferation of Epstein-Barr virus (EBV)-infected T- or NK-cells. Over time, sCAEBV progresses to high-grade lymphoma or leukemia. Most reported cases have been concentrated in Japan and China; however, the incidence remains exceedingly rare, with an annual occurrence rate of approximately 20 cases in Japan^1^.

Persistent inflammation caused by the activation of EBV-infected cells leads to progressive multi-organ failure and in severe cases, hemophagocytic lymphohistiocytosis (HLH), a life-threatening condition^2^. In addition, EBV-infected T- or NK-cells progressively acquire higher malignant potential and may eventually evolve into EBV-positive T/NK-cell lymphoma during the clinical course. Hematopoietic stem cell transplantation (HSCT) is the only curative treatment for systemic chronic active EBV disease (sCAEBV)^3^. Although the efficacy of chemotherapy and ruxolitinib, a JAK inhibitor, have been investigated, none of these approaches have succeeded in eradicating EBV-infected cells^3,4^. Thus, the development of a definitive therapeutic agent remains an urgent challenge. The disease activity of sCAEBV is manifested by the symptoms such as fever, elevated alanine aminotransferase (ALT), uveitis, progressive skin lesions, or vasculitis^5^. Disease activities at the initiation of pretransplant conditioning is a poor prognostic factor of post-transplant outcomes^6^. To improve the outcomes of transplantation, we need agents that suppress inflammation prior to HSCT.

BCL2, a member of the BCL2 family of proteins, plays a critical role in regulating mitochondrial apoptotic signaling. It functions as an anti-apoptotic factor and is implicated in the development of various hematopoietic malignancies, including chronic lymphocytic leukemia (CLL)^7^, acute myeloid leukemia (AML)^8^, and other lymphomas^9^. High levels of BCL2 support the survival and proliferation of neoplastic cells by inhibiting pro-apoptotic proteins such as BAX and BAK. Venetoclax, a selective BCL2 inhibitor, disrupts the interaction between BCL2 and BAX/BAK, thereby allowing the pro-apoptotic proteins to damage the mitochondrial outer membrane. This damage leads to the release of cytochrome c and the activation of caspase-3, ultimately inducing apoptosis. Venetoclax is approved globally for the treatment of CLL and AML. Furthermore, the reports on venetoclax in association with T/NK-cell malignancies have increased recently^10,11^. Given these observations, we investigated BCL2 expression in cells derived from sCAEBV patients and evaluated the therapeutic potential of venetoclax for this disease.

## Results

### BCL2 expression and the effects of its inhibitor venetoclax on EBV-positive T- and NK-cell lines

**Fig. 1:**
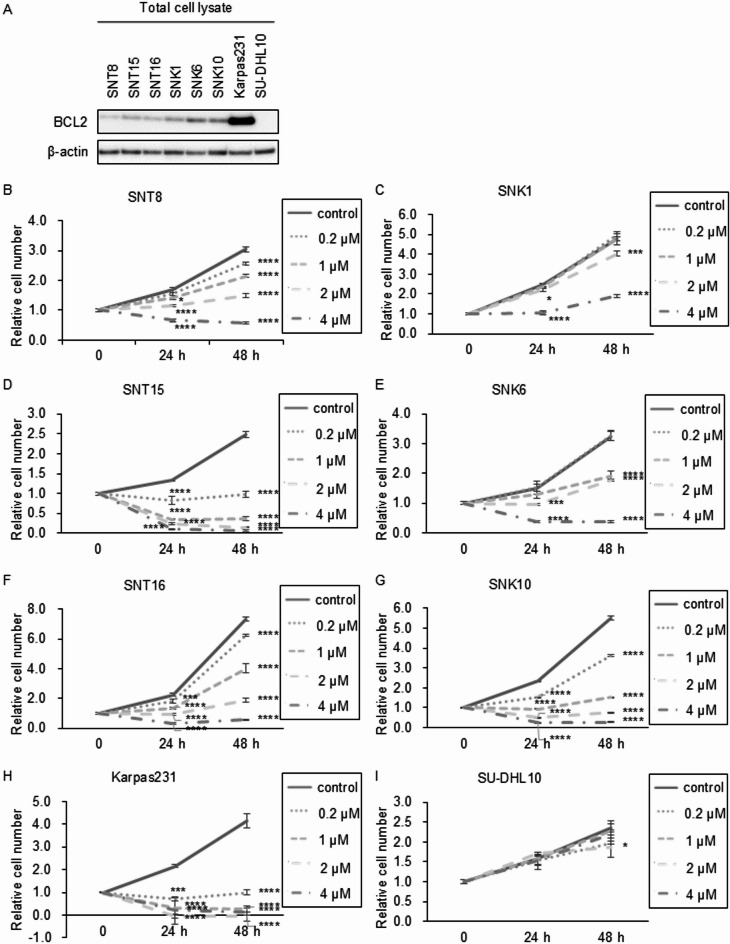
The expression of BCL2 and the effect of venetoclax on EBV-positive T- or NK-cell lines. (**A**) The expression of BCL2 protein was detected in EBV-positive T- or NK-cell lines (SNT8, SNT15, SNT16, SNK1, SNK6 and SNK10). Karpas231 and SU-DHL10 were positive and negative control of BCL2, respectively. (**B**–**I**) EBV-infected T- or NK-cell lines and the control cell lines were treated with 0, 0.2, 1, 2 and 4 µM venetoclax as indicated, for 24 and 48 h, and the number of viable cells was estimated by an MTT assay and expressed in arbitrary units. The data are shown as mean ± standard deviations (SD) of three independent experiments. At each time point, 24 and 48 h after drug stimulation, the results for the control and each drug concentration were compared using ANOVA (Dunnett’s multiple comparisons test). * for *p* < 0.05, ** for *p* < 0.01, *** for *p* < 0.001 and **** for *p* < 0.0001.

First, we assessed BCL2 expression in six EBV-positive T- and NK-cell lines using western blotting. BCL2 expression was detected in all six cell lines. In the EBV-negative B-cell line Karpas231, which is BCL2-positive, clear BCL2 expression was detected. In contrast, no BCL2 band was observed in the BCL2-negative cell line SU-DHL10 (Fig. 1A, Supplementary Figs. S1A,B). BCL2 expression levels were quantified by densitometric analysis of the western blot bands and were presented in Supplementary Figure S2A. Next, we evaluated the effects of the BCL2 inhibitor venetoclax on these cell lines. The concentrations used in this study were based on plasma levels observed in patients^12,13^. Venetoclax reduced the numbers of viable cells in a dose-dependent manner (Fig. 1B–G). The inhibitory effects of venetoclax on the viable cell numbers of EBV-infected T- or NK-cell lines with the half-inhibitory concentration (IC50) were shown in Supplementary Figs. 3 A-L. The vertical axis represented the percentage of cell count inhibition (InH (%)).

Venetoclax-induced suppression of viable cell numbers was also observed in the EBV-negative B-cell line Karpas231, which exhibited high BCL2 expression (Fig. 1H). The half-inhibitory concentration for this cell line with venetoclax was also low (Supplementary Figs. S3M,N). In contrast, no such effect was observed in the EBV-negative B-cell line SU-DHL10, which lacks BCL2 expression (Fig. 1I). The IC50 could not be calculated due to the low efficacy of venetoclax (Supplementary Figures S3O-P). These results suggest that the effect of venetoclax is BCL2-dependent.

**Fig. 2:**
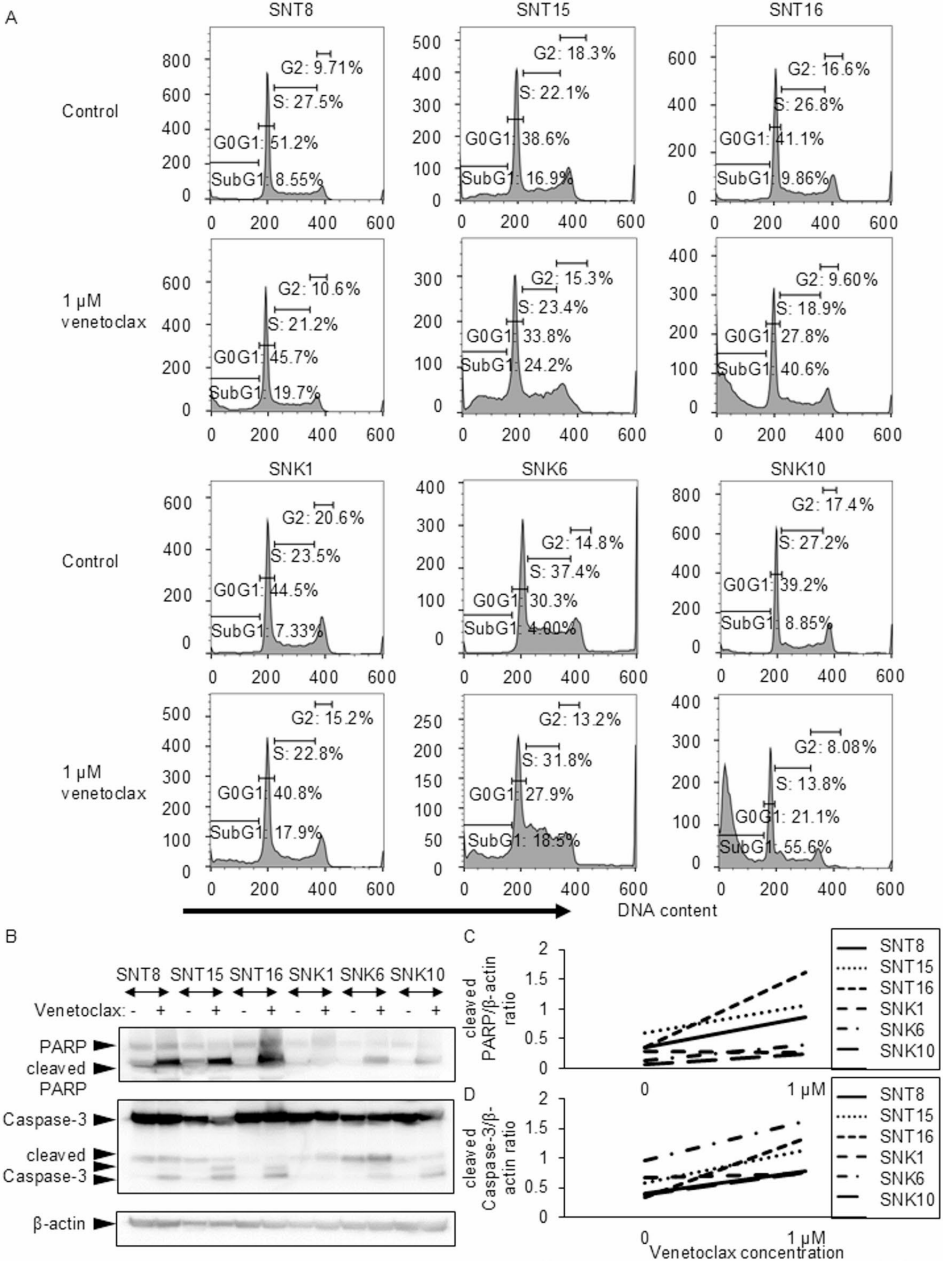
The effect of venetoclax on EBV-positive T- or NK-cell lines’ apoptosis. (**A**) EBV-infected T- or NK-cell lines were treated with venetoclax for 24 h as indicated and then analyzed. Cells were stained with Krishan’s reagent and subsequently analyzed by flow cytometry. (**B**) The fragmentation of PARP and Caspase-3 in EBV-positive T- or NK-cell lines before or after stimulation with 1 µM venetoclax was detected by western blotting analysis. (**C**,**D**) The dose-response curves for apoptosis markers (**C**: cleaved PARP, **D**: cleaved Caspase-3). The densitometry ratios relative to β-actin for (Fig. 2B).

We further examined its effects on cell cycle. At the concentrations below the maximum plasma concentration, venetoclax increased the SubG1 fraction in all six EBV-positive T- and NK-cell lines (Fig. 2A). We then examined the cleavage of PARP and caspase-3, which are both the markers of apoptosis induction. In Fig. 2B and Supplementary Figures S4A–C, the levels of cleaved PARP and cleaved caspase-3 before and after venetoclax treatment were assessed by western blotting. Figure 2C and D show densitometric quantification of these proteins, normalized to the endogenous control β-actin. As shown in these figures, venetoclax treatment led to increased levels of both cleaved PARP and cleaved caspase-3. These findings indicate that venetoclax suppresses cell survival and induces apoptosis in EBV-positive T- and NK-cell lines.

### BCL2 expression in EBV-infected cells of sCAEBV patients

**Fig. 3:**
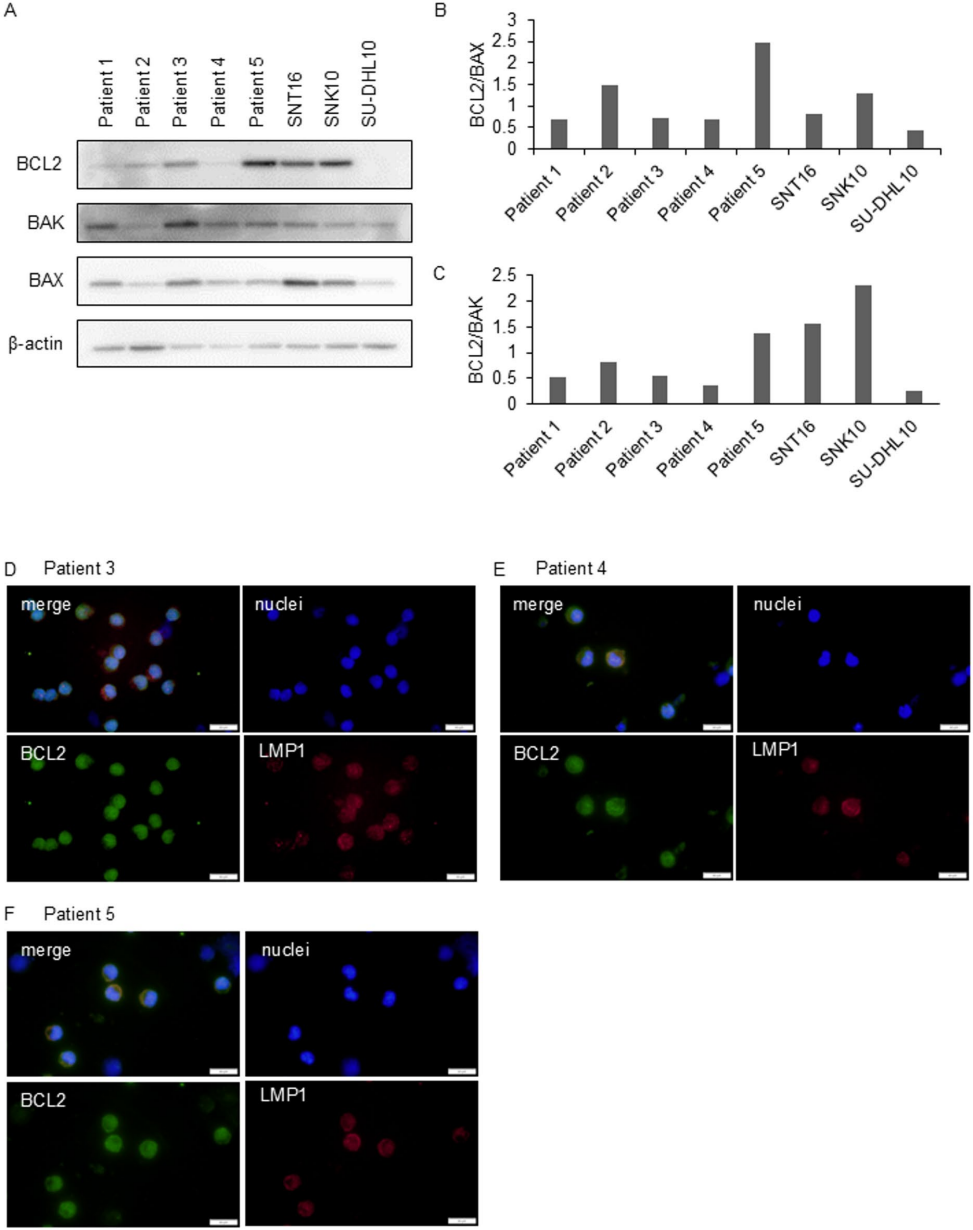
The expression of BCL2 in sCAEBV patients’ EBV-infected cells. (**A**) The expression of BCL2, BAK, and BAX was detected by western blotting analysis in five sCAEBV patients’ PBMCs. SNT16 and SNK10 were positive controls and SU-DHL10 was a negative control. (**B**,**C**) The densitometry ratio of BCL2/BAX (**B**) or BCL2/BAK (**C**) relative to β-actin for Fig. 3A was quantified by ImageJ. (**D**–**F**) The expression and the localization of BCL2, LMP1 in patients’ PBMCs was confirmed by immunofluorescence staining. Scale bar showed 20 μm.

We validated the findings from the cell line assays using PBMCs from five sCAEBV patients, which included clonally proliferating EBV-infected T- or NK-cells. The characteristics of the patients are listed in Table 1. BCL2 expression in the patients’ PBMCs was confirmed by western blotting (Fig. 3A, Supplementary Figure S5A). Then, we examined whether EBV-infected cells expressed BCL2 by immunofluorescence staining. We analyzed three patients whose samples were sufficient for more detailed investigation. The infected cells in the three patients were CD4 + cells (Patient 3), CD8 + cells (Patient 4), and CD56 + cells (Patient 5). In Fig. 3D–F, immunofluorescence staining was used to investigate BCL2 expression in EBV-infected cells. We observed that LMP1-positive cells, which represent EBV-infected cells, expressed BCL2.

**Table 1:** sCAEBV patients characteristics.

Patient no.	Sex	Age	The phenotypes of EBV-Infected cells	Clonality of the EBV-infected cells	Clinical findings	Disease activity	EBV-DNA load of whole blood (copies/µg DNA)	EBV-DNA load of the EBV-infected cell fraction (copies/µg DNA)	The ratio of EBV-infected cells in PBMCs (%)
1	M	35	CD56- NK-cell	Monoclonal	fever, liver dysfunction	active	4.9 × 10^5^	1.7 × 10^4^	70.4
2	M	27	CD4 + T-cell	Monoclonal	Fever	active	3.9 × 10^4^	1.7 × 10^5^	29.4
3	M	38	CD4 + T-cell	Monoclonal	fever, pericarditis, progressive skin lesions	active	4.3 × 10^6^	1.1 × 10^6^	86.5
4	M	50	CD8 + T-cell	Monoclonal	hemophagocytic lymphohistiocytosis	active	1.1 × 10^6^	2.7 × 10^6^	88.0
5	M	34	CD56 + NK-cell	Monoclonal	liver dysfunction	active	2.7 × 10^5^	2.4 × 10^5^	38.0

*F* female, *M* male, *PBMCs* peripheral blood mononuclear cells.

### The effects of venetoclax on PBMCs from sCAEBV patients

**Fig. 4:**
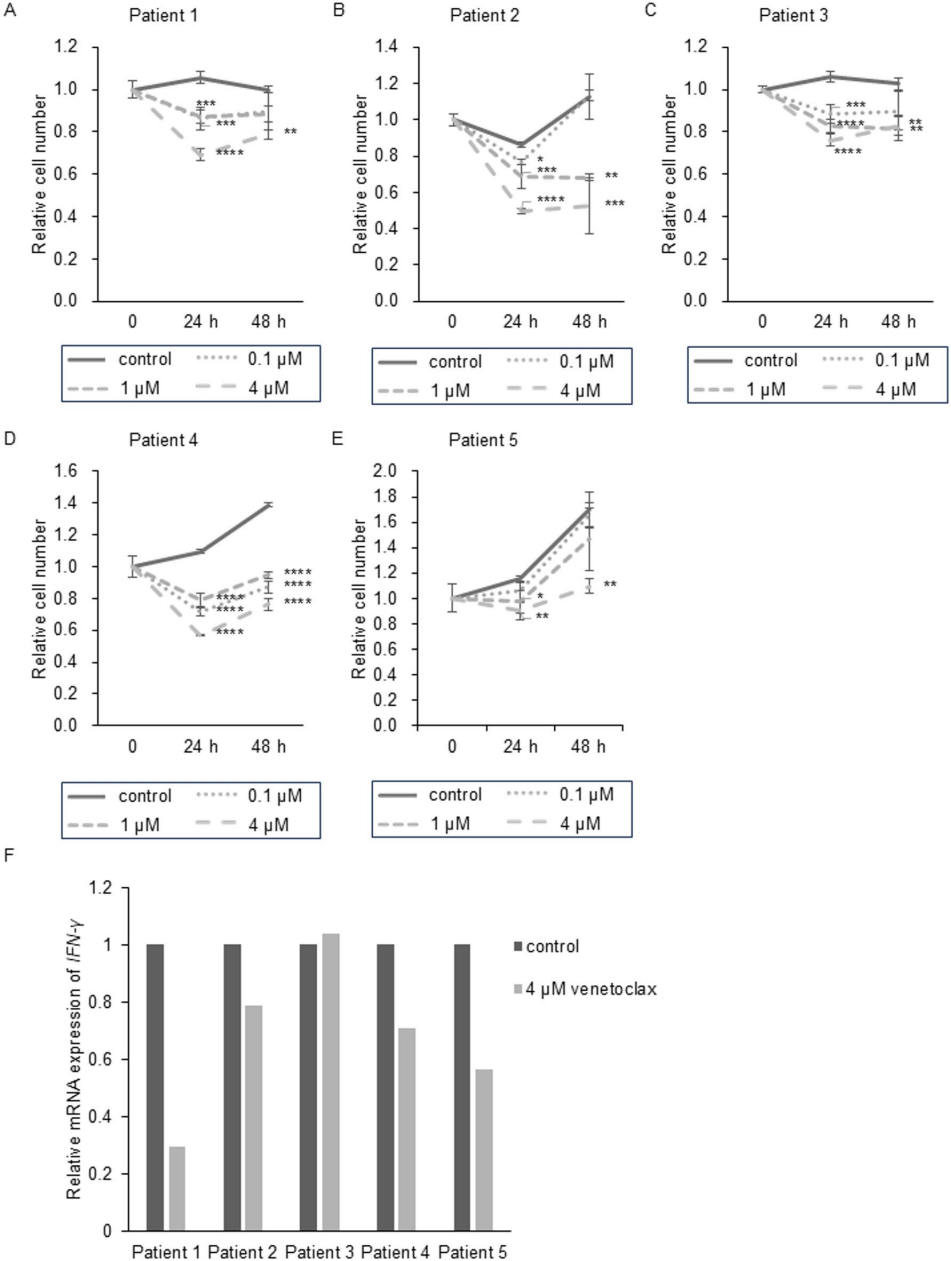
The effect of venetoclax on sCAEBV patients’ PBMCs survival and inflammatory cytokine expression. (**A**–**E**) PBMCs from five sCAEBV patients were treated with 0, 0.1, 1 and 4 µM venetoclax as indicated, for 24 and 48 h, and the number of viable cells was estimated by an XTT assay and expressed in arbitrary units. The data are shown as mean ± SD of three independent experiments. At each time point, 24 and 48 h after drug stimulation, the results for the control and each drug concentration were compared using ANOVA (Dunnett’s multiple comparisons test). * for *p* < 0.05, ** for *p* < 0.01, *** for *p* < 0.001 and **** for *p* < 0.0001. (**F**) PBMCs from five sCAEBV patients were treated with venetoclax for 24 h. The RNA was extracted and subjected to quantitative PCR assays of IFN-γ.

We investigated the effect of venetoclax on patient-derived PBMCs. The number of viable cells is shown in (Fig. 4A–E). The inhibitory effect of venetoclax on the viable PBMCs of sCAEBV patients, along with the IC50, is presented in Supplementary Figures S6A-J. Venetoclax reduced the number of viable PBMCs in all five patients significantly (Fig. 4A–E), although the IC50 values tended to be higher than those observed in cell lines. Venetoclax induces apoptosis by dissociating pro-apoptotic proteins BAX and BAK from BCL2, to which they are normally bound. To explore factors determining venetoclax sensitivity, we analyzed BAK and BAX expression and calculated BCL2/BAX and BCL2/BAK ratios in patient PBMCs (Fig. 3A–C). However, the correlation was not observed clearly between these ratios and the effect of venetoclax.

Next, we assessed whether venetoclax suppressed inflammation or not. We analyzed the mRNA expression of the inflammatory cytokine IFN-γ, which is significantly elevated in the plasma of sCAEBV patients compared to healthy individuals^14^. Venetoclax downregulated IFN-γ mRNA expression in all patients except Patient 3 (Fig. 4F). It is reported that STAT3, which controls the expression of IFN-γ, is constitutively activated in EBV-infected cells of sCAEBV^15^. We therefore investigated the effect of venetoclax treatment on STAT3 activation. As shown in Supplementary Figures S7A,B, the inhibition of STAT3 activation by venetoclax was not observed clearly.

### Venetoclax may suppress the engraftment of EBV-positive cells in a xenograft model

**Fig. 5:**
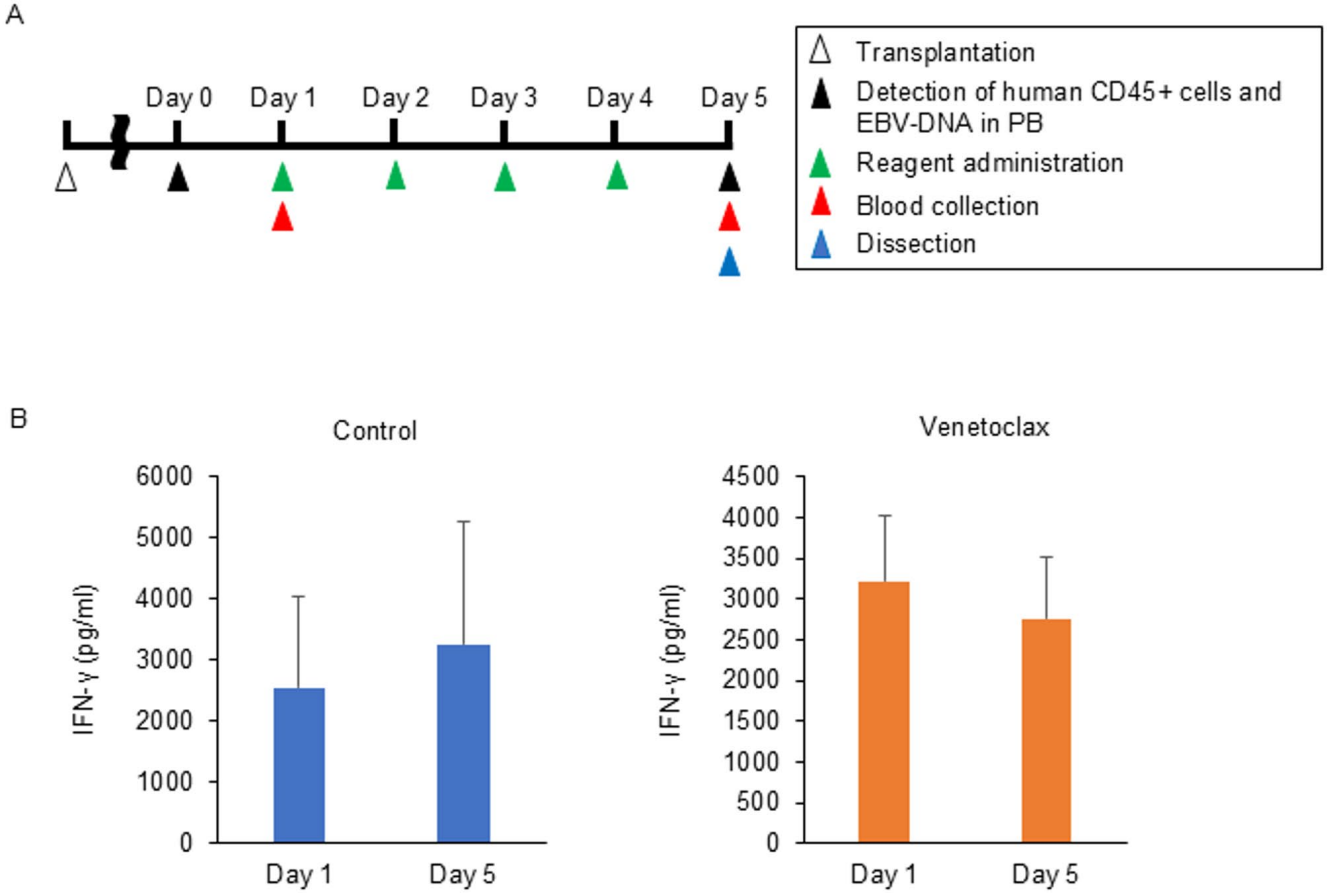
The anti-inflammatory effect of venetoclax on sCAEBV xenograft model. (**A**) The schedule of transplantation and drug administration. (**B**) The average of plasma concentration of IFN-γ was compared between Day 1 and Day 5 in the same treatment, control or venetoclax. The data were shown as mean ± SD (control: *n* = 7, venetoclax: *n* = 8).

Finally, we investigated the in vivo effects of venetoclax by sCAEBV xenograft models. Model mice were generated by transplanting PBMCs from Patients 2, 3, and 5, who provided sufficient cells required for the transplantation. First, we evaluated the anti-inflammatory effects of venetoclax. The schedule of transplantation and drug administration are shown in (Fig. 5A). When human CD45-positive cells and EBV-DNA were detected in the peripheral blood (PB), the treatment with either venetoclax or the solvent was initiated. Engraftment was successful in 50% of the patient-derived xenograft models. As an indicator of inflammation, plasma IFN-γ levels were measured. As shown in Fig. 5B, venetoclax-treated mice exhibited a nonsignificant trend toward reduced IFN-γ levels, whereas the reduction was not observed in solvent-treated controls.

**Fig. 6:**
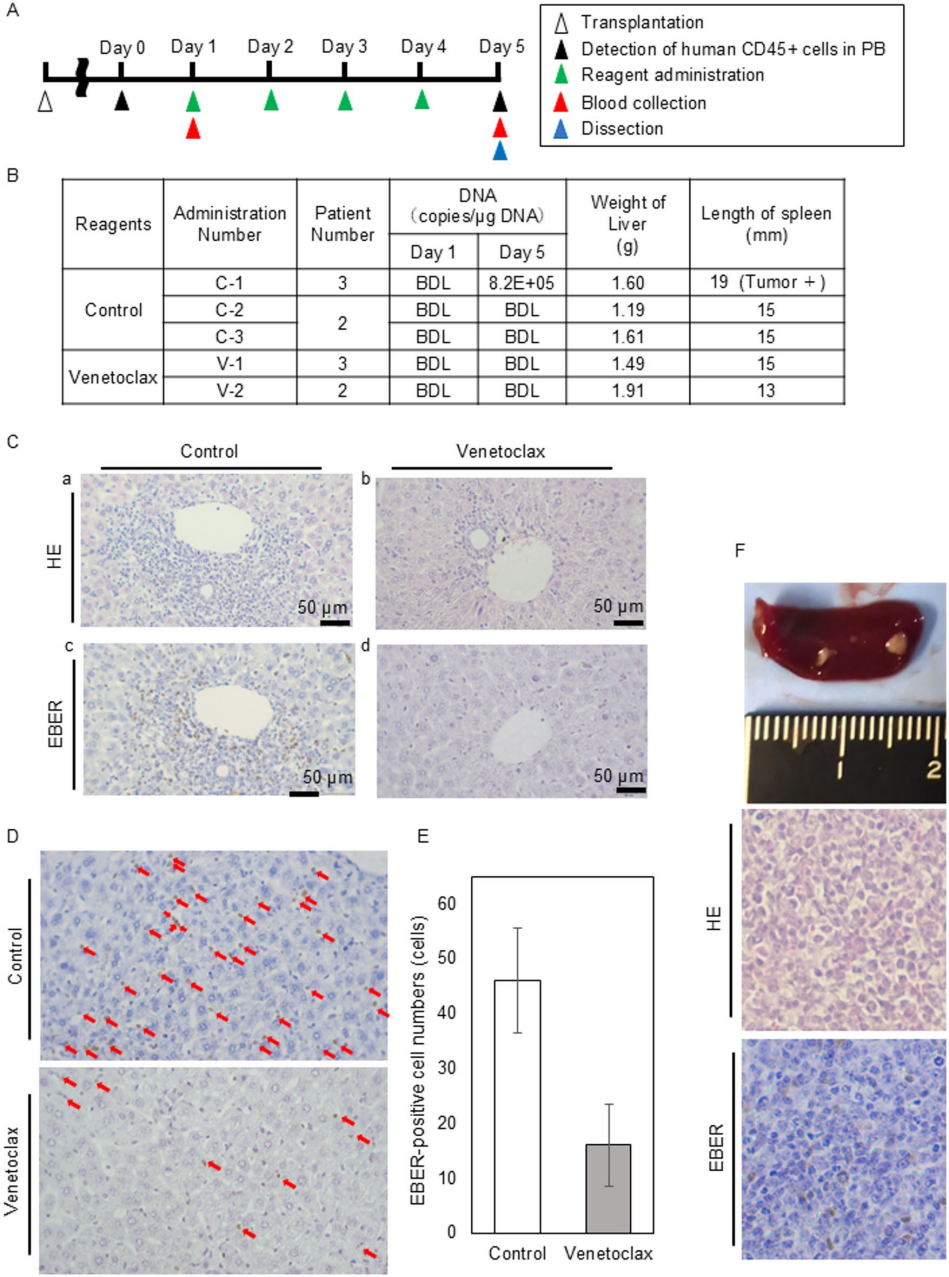
The effect of venetoclax on the engraftment of sCAEBV patients’ PBMCs in the xenograft model. (**A**) The schedule of transplantation and drug administration. (**B**) The findings in venetoclax-treated mice and the solvent-treated mice. *BDL* Below the detection limit. (**C**) Representative images of hematoxylin/eosin (HE) staining and EBER-ISH are shown after four doses treatment of venetoclax (**b**,**d**) or the solvent (**a**,**c**). Sections of portal area of liver were stained with HE-staining (**a**,**b**) and EBER-ISH (**c**,**d**). Scale bars indicate 50 μm. (**D**) Representative images of liver sinusoids of EBER-ISH are shown after four doses treatment of venetoclax or the solvent-treated mice. The red arrows point to EBV-infected cells infiltrated into sinusoids. The original magnification was × 400. (**E**) The mean number of EBV infected (EBER-positive) cells in liver sinusoids was counted at three selected sites in EBER-ISH images and compared in control- and venetoclax-treated mice. *p < 0.05. (**F**) Tumor formation in the spleen of a solvent-treated mouse. The pathological images of the tumor stained with HE-staining and EBER-ISH. The original magnification was × 400.

Next, we analyzed the effects of venetoclax on the engraftment of EBV-positive cells. The schedule of transplantation and drug administration are shown in (Fig. 6A). PBMCs from Patients 2 and 3 were transplanted to mice following the previous protocol. Mononuclear cells in PB were monitored weekly by flow cytometry. When human CD45-positive cells were detected in PB for two consecutive weeks, we acknowledged the engraftment, and the treatment with either venetoclax or the solvent was initiated.

The engraftment of EBV-positive cells was assessed by detecting EBV-DNA in PB and observing the infiltration of EBV-infected cells in organs. At the start of treatment, no EBV-DNA was detected in PB. Five mice were included: two received venetoclax for four days, and three served as controls receiving the solvent. On Day 5, mice were dissected. As shown in Fig. 6B, none of the venetoclax-treated mice exhibited detectable EBV-DNA in PB, whereas one control mouse (C-1) did.

The results of the pathological analysis are shown in (Fig. 6C–G). As shown in Fig. 6C and D, EBV-positive cells infiltrated the perisinusoidal regions of the liver in solvent-treated mice, whereas such infiltration was scarcely observed in venetoclax-treated mice. EBV-positive sinusoidal infiltrates were quantified and are shown in Fig. 6E. The mean number of EBV infected cells in liver sinusoids was counted at three selected sites in EBER-ISH images and compared in mice C-1 and V-1. The number of EBV-infected cells in the liver was significantly smaller in V-1 compared to C-1. Figure 6F shows the spleen of mouse C-1 treated with solvent. The spleen was swollen and contained solid tumor where the infiltration of EBV-infected cells was observed. None of the venetoclax-treated mice developed tumors.

## Discussion

In this study, we demonstrated that venetoclax exerts both anti-tumor and anti-inflammatory effects, implying its unique therapeutic potential in the treatment of sCAEBV. We confirmed the BCL2 expressions in EBV-infected T- and NK-cell lines and in EBV-infected cells within PBMCs derived from sCAEBV patients. Venetoclax reduced cell viability and induced apoptosis in both cell lines and in patient-derived PBMCs. It also prevented the engraftment of EBV-infected T-cells in xenograft models. Given that BCL2 supports the survival of hematopoietic tumor cells, these findings suggest that venetoclax directly targets EBV-infected cells in sCAEBV. However, the pathogenesis of sCAEBV involves not only EBV-infected cells but also EBV-uninfected cells that contribute to the cellular network. Notably, our previous research demonstrated that CD4-positive cells, regardless of EBV infection status, are essential for the engraftment of EBV-infected T- or NK-cells in sCAEBV mouse models¹⁴. BCL2 expression was observed even when the proportion of EBV-infected cells was low, indicating its presence in both infected and uninfected cells. Venetoclax may therefore act on multiple cell populations that are critical to disease progression. Interestingly, in experiments using patient-derived PBMCs—particularly in the cases where the proportion of EBV-infected cells was low (e.g., Patients 2 and 5)—the total PBMC count initially decreased following venetoclax treatment but subsequently increased. This observation may reflect the heterogeneity of lymphocyte subsets. Because patient-derived PBMCs contain a mixture of EBV-infected and uninfected cells, their responses to venetoclax may vary accordingly. Further investigation into the phenotypic and functional characteristics of these constituent cells before and after venetoclax treatment is needed to avoid excessive immunosuppression.

What are the molecular mechanisms underlying venetoclax’s suppression of cell proliferation and IFN-γ production? BCL2, the molecular target of venetoclax, plays a central role in promoting cell survival by binding to and inhibiting the pro-apoptotic proteins BAX and BAK, which would otherwise initiate mitochondrial apoptosis. Venetoclax disrupts this interaction, resulting in the release of BAX and BAK, mitochondrial outer membrane permeabilization, cytochrome c release, caspase-3 activation, and ultimately apoptosis. Expression of both BAX and BAK was confirmed in all patient-derived cells. However, compared with cell lines, venetoclax exhibited higher IC50 values and tended to have weaker effects in patient PBMCs. This reduced sensitivity may be attributed to the heterogeneous nature of PBMCs, which include non-infected bystander cells in addition to EBV-infected ones. Meanwhile, the lack of correlation between BCL2 expression levels relative to BAX and BAK and the suppression of cell viability suggests that molecules other than BCL2 may also contribute to cell survival. One such candidate is STAT3, which we previously reported to be constitutively activated in EBV-positive T- and NK-cells in sCAEBV and to promote their survival. Although patient samples were not available for validation, STAT3 was not suppressed by venetoclax in the cell lines, suggesting that STAT3-mediated survival pathways may function independently of BCL2 in sCAEBV. STAT3 also contributes to the production of IFN-γ, an inflammatory cytokine that is elevated in the plasma of patients with sCAEBV, and plays a role in establishing the inflammatory pathology of the disease. In this study, we also demonstrated that venetoclax suppresses IFN-γ production. To our knowledge, this mechanism of venetoclax action has not been previously reported in sCAEBV. However, given that venetoclax did not suppress STAT3 activation in the cell lines, it is possible that the pathway responsible for IFN-γ production is independent of STAT3. In Patient 3, who did not show a clear IFN-γ–suppressive response to venetoclax, STAT3 may have played a central role in IFN-γ production. Recently, we showed that ruxolitinib, a JAK1/2 inhibitor, suppresses STAT3 activation and IFN-γ production in vitro and improves disease activity and systemic inflammation in sCAEBV patients^4,15^. Since venetoclax and ruxolitinib likely target distinct but complementary pathways, their combination may provide more effective suppression of EBV-positive T- and NK-cell survival and IFN-γ–driven inflammation in sCAEBV. Future studies should focus on elucidating the downstream signaling pathways of BCL2 inhibition that regulate cytokine production.

We observed the upregulation of cleaved caspase-3 in six EBV-positive T- and NK-cell lines following venetoclax treatment. This indicates the involvement of the caspase-3 pathway in venetoclax-induced apoptosis and is supported by the concomitant increase in cleaved PARP expression. However, in SNK1 cells, cleaved caspase-3 expression was low despite an increased sub-G1 population. The low expression suggests the involvement of caspase-independent pathways. Apoptosis-inducing factor (AIF) is associated with caspase-independent apoptosis. The result of our unpublished analysis shows increased AIF expression in venetoclax-treated SNK1 cells, indicating the possibility of venetoclax-induced apoptosis involving both caspase-dependent and -independent mechanisms.

This study has several limitations; the rarity of sCAEBV and limited patient sample availability which constrained both in vitro analyses and model development. The safety of long-term venetoclax administration, particularly its immunosuppressive effects due to lymphocyte reduction, must be also carefully assessed. Since venetoclax may affect uninfected lymphocytes, determining the optimal dosage, treatment duration, and long-term safety profile in sCAEBV is crucial to minimize immunosuppression.

In addition to BCL2, other BCL2 family members, such as BCL-xL and MCL1, may also contribute to the survival of EBV-infected cells and are worth examining in the future. Notably, Liu et al. have identified BCL-xL as a potential therapeutic target in extranodal NK/T-cell lymphoma, suggesting broader therapeutic relevance of this protein family^16^. Our findings demonstrate that venetoclax exerts potent anti-tumor and anti-inflammatory effects in vitro and in vivo, underscoring its potential as a novel therapeutic agent for sCAEBV.

## Materials and methods

### Cells and reagents

We used EBV-positive T-cell lines (SNT8, SNT15, SNT16) and EBV-positive NK-cell lines (SNK1, SNK6, SNK10), which were established from EBV-positive T- or NK-cell lymphoid neoplasms^17–19^. These cell lines were cultured in Artemis medium-2 (Nihon Techno Service Co., Ltd., Ibaraki, Japan). As controls, we used two EBV-negative B-cell lines derived from diffuse large B-cell lymphoma (DLBCL): Karpas231, characterized by high BCL2 expression due to IGH-BCL2 fusion (t(14;18)(q32;q21)), obtained from ECACC (Salisbury, UK), and SU-DHL10, which lacks BCL2 expression, sourced from ATCC (Manassas, VA, USA). Both B-cell lines were cultured in RPMI-1640 medium supplemented with 20% FBS.

Venetoclax, a BCL2 inhibitor, was purchased from Selleck Chemicals (Houston, USA). For western blotting, we used the following antibodies: anti-BCL2 (Santa Cruz Biotechnology, Dallas, USA), anti-BAK (Cell Signaling Technology, Danvers, USA), anti-BAX (Cell Signaling Technology), anti-PARP (Cell Signaling Technology), anti-Caspase-3 (Santa Cruz Biotechnology), anti-phospho-Stat3 (Tyr705) (Cell signaling Technology), anti-phospho-Stat3 (Ser727) (Cell signaling Technology), anti-Stat3 (Santa Cruz Biotechnology) and anti-β-actin (Merck KGaA, Darmstadt, Germany). For immunofluorescence staining, we used anti-BCL2 (Proteintech, Sankt Leon-Rot, Germany) and anti-LMP1^20^ antibodies. Secondary antibodies included anti-rabbit IgG (H + L) Alexa Fluor Plus 488 and anti-mouse IgG (H + L) Alexa Fluor Plus 555, both from Thermo Fisher Scientific (Waltham, USA).

### Western blotting

The assay was performed as described previously^20,21^. For western blotting, lysates were prepared by dissolving in lysis buffer to a concentration of 1 × 10^7^ cells/ml, and equal amounts of lysates (5 µl) were loaded onto each lane. The densitometry ratios were quantified by ImageJ (National Institutes of Health, Bethesda, USA).

### Detection of cell viability and apoptosis

All venetoclax-stimulated cell experiments were performed at a cell concentration of 5 × 10^5^ cells/ml. After stimulation, the numbers of viable cells were measured using XTT (Cell Count Reagent SF, Nacalai Tesque, Inc., Kyoto, Japan) and MTT (MTT Cell Count Kit, Nacalai Tesque, Inc.) assays following the manufacturer’s instructions. The 50% cell viability by venetoclax was calculated for each cell line or patients’ PBMCs at each time point (24 and 48 h). In particular, raw absorbance of each treated was normalized to the untreated control (0 µM). Then, the inhibition of cell numbers (InH (%)) in the control (0 µM) was defined as 0%, and the concentration at which venetoclax inhibited cell viability by 50% (IC50) was calculated using GraphPad Prism and Excel. The vertical axis represents the inhibition rate of cell numbers. Apoptosis was detected by treating cells with Krishan’s reagent (0.05 mg/ml propidium iodide, 0.1% sodium citrate, 0.02 mg/ml ribonuclease A, and 0.3% NP-40) and analyzing by flow cytometry^22^. The fraction ratio of apoptotic cells was calculated using FlowJo software (BD Biosciences, Franklin Lakes, USA).

### Diagnosis of sCAEBV

sCAEBV patients were diagnosed based on the following four criteria^3^: (1) infectious mononucleosis-like symptoms persisting for three months or longer (continuous or intermittent), (2) increased EBV genome levels in peripheral blood or diseased tissue, (3) EBV infection of T- or NK-cells, and (4) exclusion of other known diseases (CAEBV Research Group of the Ministry of Health, Labour and Welfare of Japan, 2015). These criteria align with the definition of CAEBV in the 2017 WHO classification^23^.

### Patient selection

Samples used in the experiments were obtained from untreated patients with active disease. Disease activity was defined by the presence of at least one of the following clinical features: fever, elevation of ALT to more than twice the institutional upper limit, uveitis, or vasculitis, as previously described by Yonese et al.^3^.

### Immunofluorescence staining

PBMCs were adhered to slides by centrifugation using Cyto-Tek^®^ 2500 (Sakura Finetek Japan Co., Ltd., Tokyo, Japan). The cells were fixed on ice for 10 min using a mixture of acetone (Fujifilm Wako Pure Chemical Corporation, Osaka, Japan) and methanol (Merck KGaA, Darmstadt, Germany) and then blocked with BLOCK ACE Powder solution (Megmilk Snow Brand Co., Ltd., Tokyo, Japan) at room temperature for one hour. The slides were incubated with the aforementioned antibodies. Nuclei were stained with ProLong Gold Antifade Reagent with DAPI (Thermo Fisher Scientific, Waltham, USA). Images were captured using a CKX53 Cell Culture Microscope (Olympus Corporation, Tokyo, Japan).

### qRT-PCR analysis

EBV-DNA loads in whole blood were quantified by real-time polymerase chain reaction (RT-PCR) as previously described^24^. Owing to the sensitivity of qRT-PCR analysis, samples that did not exhibit an increase in the amplification curve were classified as ND (Not determined). For mRNA detection of *IFN-γ* and *GAPDH*, we used TaqMan^®^ Gene Expression Assay primers: *IFN-γ* (Hs00989291_m1) and *GAPDH* (Hs99999905_m1) (Thermo Fisher Scientific).

### Xenograft model generation

Six-week-old male NOD/Shi-scid/IL-2Rγnull (NOG) mice were obtained from the Central Institute for Experimental Animals (Kawasaki, Japan) and were maintained under specific pathogen-free conditions. sCAEBV model mice were generated by injecting PBMCs (1–2 × 10⁷ cells/mL) from sCAEBV patients through the tail vein, as previously reported^14,25^.

### In vivo venetoclax treatment

Venetoclax was prepared in a solvent consisting of 5% DMSO, 50% polyethylene glycol 300 (PEG300), and 5% Tween80 in ddH₂O, following the manufacturer’s instructions. Venetoclax was administered to mice at a dose of 50 mg/kg via daily oral gavage for four days^26,27^. The same volume of the solvent was administered to the control group.

### EBER-in situ hybridization

For EBER in situ hybridization (EBER-ISH), tissues were hybridized with a hybridization solution (Merck KGaA) and an [EBER] PNA Probe/Dako Fluorescein (Agilent Technologies, Santa Clara, USA) at 55 °C for 90 min. The samples were then stained with Molecular Probes Fluorescein (Thermo Fisher Scientific) and DAB.

### Plasma concentration of IFN-γ in vivo

IFN-γ concentration in plasma was detected by human IFN-γ quantikine ELISA kit (R&D Systems) as manufacturer’s instructions.

### Statistical analysis

Data are presented as the mean ± standard deviation (SD). Statistical analyses were performed using Student’s two-tailed t-test, ANOVA with Dunnett’s multiple comparisons test and Two-way repeated measures ANOVA with GraphPad Prism 6 and 8 (GraphPad, San Diego, USA).

### Ethics approval statement

The studies involving human participants were performed in accordance with the Declaration of Helsinki. They were reviewed and approved by the ethical committees of St. Marianna University School of Medicine (4709) and the Institute of Science, Tokyo (G2000-176). Written informed consent was obtained from all participating patients prior to their involvement in the study. Animal experiments using NOG mice were conducted in accordance with the Guidelines for Animal Experimentation of the Japanese Association for Laboratory Animal Sciences and the ARRIVE guidelines. These experiments were approved by the Institutional Animal Experimentation Committee of the Institute of Science, Tokyo (A2023-158 A).

## Electronic supplementary material

Below is the link to the electronic supplementary material.

Supplementary Material 1

Supplementary Material 2

## Data Availability

Data is available from the corresponding author upon reasonable request.
