# Supplementary material for: Effects of venetoclax, a BCL2 inhibitor, in systemic chronic active Epstein-Barr virus disease
Source: Sci Rep. 2025 May 27;15:18569. doi: 10.1038/s41598-025-03719-9 (PMC12117165; doi:10.1038/s41598-025-03719-9)
Supplement: Supplementary file 1 — Supplementary Material 1 [file 41598_2025_3719_MOESM1_ESM.docx]

**Supplementary Figure Legends**

**Supplementary Figure S1. The full-length gel of Figure 1A.**

1. The original, uncropped Western blot image for BCL2 shown in Figure 1A.
2. The original, uncropped Western blot image for β-actin in Figure 1A.

**Supplementary Figure S2. The densitometry ratios of Figure 1A.**

(A) The densitometry ratio of BCL2 relative to β-actin of Figure 1A was quantified by ImageJ.

**Supplementary Figure S3. The inhibitory effect of venetoclax on the viable cell numbers of EBV-infected T- or NK-cell lines.**

IC50 was calculated for each cell line at two time points (24 and 48 hours). The inhibition of cell numbers in the control (0 µM) was defined as 0%, and the concentration at which venetoclax inhibited cell numbers by 50% was calculated. The vertical axis represents the percentage of cell count inhibition (InH (%)). The half-inhibitory concentration (IC50) was calculated using GraphPad Prism and Excel.

(A, B) IC50 value of vanetoclax in SNT8 in Figure 1B.

(C, D) IC50 value of vanetoclax in SNT15 in Figure 1D.

(E, F) IC50 value of vanetoclax in SNT16 in Figure 1F.

(G, H) IC50 value of vanetoclax in SNK1 in Figure 1C.

(I, J) IC50 value of vanetoclax in SNK6 in Figure 1E.

(K, L) IC50 value of vanetoclax in SNK10 in Figure 1G.

(M, N) IC50 value of vanetoclax in Karpas231 in Figure 1H.

(O, P) IC50 value of vanetoclax in SU-DHL10 in Figure 1I.

**Supplementary Figure S4. The full-length gel for Figure 2B.**

1. The original, uncropped western blot image for PARP in Figure 2B.
2. The original, uncropped western blot image for Caspase-3 in Figure 2B.
3. The original, uncropped western blot image for β-actin in Figure 2B.

**Supplementary Figure S5. The full-length gel for Figure 3A.**

1. The original, uncropped western blot image for BCL2 in Figure 3A.
2. The original, uncropped western blot image for BAX in Figure 3A.
3. The original, uncropped western blot image for BAK in Figure 3A.
4. The original, uncropped western blot image for β-actin in Figure 3A.

**Supplementary Figure S6. The inhibitory effect of venetoclax on the viable cell numbers of sCAEBV patients’ PBMCs.**

IC50 was calculated for each patient at two time points (24 and 48 hours). The inhibition of cell numbers in the control (0 µM) was defined as 0%, and the concentration at which venetoclax inhibited cell numbers by 50% was calculated. The vertical axis represents the percentage of cell count inhibition (InH (%)). The half-inhibitory concentration (IC50) was calculated using GraphPad Prism and Excel.

(A, B) IC50 value of vanetoclax in Patient 1 in Figure 4A.

(C, D) IC50 value of vanetoclax in Patient 2 in Figure 4B.

(E, F) IC50 value of vanetoclax in Patient 3 in Figure 4C.

(G, H) IC50 value of vanetoclax in Patient 4 in Figure 4D.

(I, J) IC50 value of vanetoclax in Patient 5 in Figure 4E.

**Supplementary Figure S7. The effect of venetoclax to the expression of phospho-STAT3.**

(A, B) The expression of phospho-STAT3 (Tyr705), phospho-STAT3 (Ser727) and STAT3 before and after the stimulation with 1 µM venetoclax was detected by western blotting analysis and quantified in the densitometry ratios relative to β-actin in (A) SNT8, SNT15, SNT16 and (B) SNK1, SNK6, SNK10.
